# 

**Published:** 1905-03

**Authors:** 


### CONTENTS.

| | PAGE |
|---|---|
| **The Prevention of Apoplexy.** By T. Clifford Allbutt, M.D., F.R.S. | 1 |
| **Some Notes on Examinations for Life Assurance.** By R. Shingleton Smith, M.D. Lond., F.R.C.P. ... ... ... ... ... | 11 |
| **Nephropexy by Means of the Application of Strong Carbolic Acid and Temporary Support, with Report of Eight Cases.** By T. Carwardine, M.S., M.B. Lond., F.R.C.S. ... ... ... | 24 |
| **On the Advantages of Enucleation of the Tonsils over their Removal by the Guillotine.** By Ernest W. Hey Groves, M.D., B.S., B.Sc. Lond. ... ... | 32 |
| **Three Cases of Ovarian Tumour complicating Pregnancy, Labour and the Puerperium respectively.** By Ewen J. Maclean, M.D., M.Ch. Edin., M.R.C.P. Lond., F.R.S. Edin. ... ... | 40 |
| **Progress of the Medical Sciences:** | |
| **Medicine.** Theodore Fisher, M.D. Lond., M.R.C.P. ... ... ... ... ... ... ... | 49 |
| **Surgery.** J. Lacy Firth, M.S., M.D. Lond. | 53 |
| **Laryngology and Rhinology.** P. Watson Williams, M.D. Lond. ... | 57 |
| **Reviews of Books** ... ... ... ... ... ... | 60 |
| **Editorial Notes** ... ... ... ... ... ... | 73 |
| **Notes on Preparations for the Sick** ... | 76 |
| **The Library of the Bristol Medico-Chirurgical Society** ... ... ... | 82 |
| **Meetings of Societies** ... ... ... ... ... | 84 |
| **Obituary Notices** ... ... ... ... ... | 92 |
| **Local Medical Notes** ... ... ... ... ... | 95 |

